# Memory and comprehension deficits in spatial descriptions of children with non-verbal and reading disabilities

**DOI:** 10.3389/fpsyg.2014.01534

**Published:** 2015-01-07

**Authors:** Irene C. Mammarella, Chiara Meneghetti, Francesca Pazzaglia, Cesare Cornoldi

**Affiliations:** ^1^Department of Developmental and Social Psychology, University of PadovaPadova, Italy; ^2^Department of General Psychology, University of PadovaPadova, Italy

**Keywords:** non-verbal learning disability, reading disability, spatial descriptions, spatial perspective

## Abstract

The present study investigated the difficulties encountered by children with non-verbal learning disability (NLD) and reading disability (RD) when processing spatial information derived from descriptions, based on the assumption that both groups should find it more difficult than matched controls, but for different reasons, i.e., due to a memory encoding difficulty in cases of RD and to spatial information comprehension problems in cases of NLD. Spatial descriptions from both survey and route perspectives were presented to 9–12-year-old children divided into three groups: NLD (*N* = 12); RD (*N* = 12), and typically developing controls (TD; *N* = 15); then participants completed a sentence verification task and a memory for locations task. The sentence verification task was presented in two conditions: in one the children could refer to the text while answering the questions (i.e., text present condition), and in the other the text was withdrawn (i.e., text absent condition). Results showed that the RD group benefited from the text present condition, but was impaired to the same extent as the NLD group in the text absent condition, suggesting that the NLD children’s difficulty is due mainly to their poor comprehension of spatial descriptions, while the RD children’s difficulty is due more to a memory encoding problem. These results are discussed in terms of their implications in the neuropsychological profiles of children with NLD or RD, and the processes involved in spatial descriptions.

## INTRODUCTION

In everyday life people continuously learn spatial relations, which can be acquired from various sources, including visual inputs (i.e., maps, navigation, etc.) and verbal information (i.e., spatial descriptions). When people learn spatial directions or landmarks from spoken or written language, they build a mental model, i.e., an internal representation that resembles the structure of the corresponding outside world (Johnson-Laird, 1983), preserving such spatial features as the relationship between landmarks (Perrig and Kintsch, 1985; Taylor and Tversky, 1992). The development of this mental model is sustained by comprehension (van Dijk and Kintsch, 1983), by underlying cognitive processes such as working memory (WM; see Gyselinck and Meneghetti, 2011 for a review) and updating (Avraamides et al., 2013), and by long-term memory for its maintenance (Shelton and McNamara, 2004). Comprehension and memory processes are rarely considered separately in the literature, however, (Cain and Oakhill, 2006), and most studies have focused on how spatial information appears and is represented in memory (Gyselinck and Meneghetti, 2011). Spatial descriptions thus conjugate both verbal aspects (referring to the format used) and spatial aspects (referring to their content). Analyzing these two aspects of learning spatial descriptions is particularly intriguing when studying individuals with weak abilities in processing and/or recalling spatial information (as suggested by Borella et al., 2014). Populations of interest include: individuals who have weaknesses in processing and/or recalling spatial material (but substantially preserved verbal skills), as in the case of children with non-verbal learning disabilities (NLD); and those with weaknesses in processing and/or recalling verbal material, such as children with reading disabilities (RD). Concentrating on children with these different profiles could therefore help us to better distinguish between the contributions of verbal and spatial aspects in forming and maintaining an environment representation.

It has often been reported that children with learning disabilities have trouble processing texts, but their difficulties seem to differ, depending on their specific type of disability. In fact, children exhibiting RD (i.e., dyslexia) are typically competent in non-verbal domains and weak in language processing and verbal recall. Partly as a consequence of their reading decoding difficulties, their more limited exposure to written texts, and their phonological loop’s low storage capacity (Gould and Glencross, 1990; Ackerman and Dykman, 1993; Palmer, 2000; Helland and Asbjørnsen, 2004), children with RD may find it difficult to remember the content of complex texts. For instance, Kramer et al. (2000) administered the Children’s Version of the California Verbal Learning Test and found that long-term memory for verbal material was impaired in children with RD: their RD group learned the list of items more slowly, recalled fewer words in the last learning trial and the delayed trials, and performed less well in the recognition condition. The authors attributed these results to RD children having a poor encoding capacity because their retention and retrieval appeared as good as in controls, suggesting that the verbal memory problems that children with RD encounter can be compensated by adequate encoding.

A symmetrical profile in the field of learning disabilities concerns children exhibiting NLD, who are competent in verbal domains and have a high verbal IQ, but are weak in non-verbal, and particularly in visuospatial domains, encountering serious adaptive and learning difficulties (Rourke, 1995). It is worth noting that the DSM-5 [American Psychiatric Association (APA), 2013] mentions only one learning disability category, and the ICD-10 [World Health Organization (WHO), 1992] did not mention NLD as a specific category. In fact, although most researchers and clinicians agree that a profile of NLD clearly exists (but see Spreen, 2011, for an exception), they disagree on the need for a separate clinical category and on the criteria to use in its identification. Various attempts have recently been made to review the literature (Fine et al., 2013) and establish appropriate diagnostic criteria (Mammarella and Cornoldi, 2014). Mammarella and Cornoldi (2014) suggested that at least some of the following criteria should be met for a child to be diagnosed with NLD: a low visuospatial intelligence and a discrepancy between visuospatial and verbal intelligence; poor visuo-constructive and fine motor skills; a discrepancy between mathematical and reading achievement, the former being worse than the latter; poor performance in visuospatial working memory (VSWM) tasks; and associated socio-emotional problems.

Previous studies focused on examining and demonstrating specific visuospatial deficits in NLD children, particularly concerning their VSWM (e.g., Cornoldi et al., 1995; Mammarella and Cornoldi, 2005a,b; Mammarella et al., 2006), vis-à-vis their good verbal abilities. But NLD children also seem to have specific linguistic deficits, especially when pragmatic or spatial aspects are involved as well (Worling et al., 1999; Humphries et al., 2004). They have trouble with inferences, specifically when spatial relationships have to be processed, giving the impression that they find it hard to develop the spatial mental models they need to make inferences correctly (Johnson-Laird, 1983).

In short, children with RD and NLD may – for different reasons – have difficulty in processing verbal descriptions that include spatial information. In an earlier study, Mammarella et al. (2009) investigated the ability to form mental models derived from spatial descriptions of 9–12-year-olds with NLD, RD, and typical development (TD). Spatial descriptions were presented from survey and route perspectives, two typical ways of representing spatial information (Taylor and Tversky, 1992; Brunye and Taylor, 2008). Route descriptions present information from the point of view of a person moving within an environment, using an intrinsic frame of reference and egocentric terms (e.g., right, left, front, and back), and they follow a linear layout given by the order in which landmarks appear along the way. Survey descriptions present information from a bird’s eye view, sometimes with a strongly hierarchical organization, and they are characterized by an extrinsic frame of reference and the use of canonical terms (e.g., north, south, east, and west; Tversky, 1991). The texts used in the study by Mammarella et al. (2009) were presented according to the procedure adopted by Meneghetti and coauthors in previous studies (see Gyselinck and Meneghetti, 2011; Meneghetti et al., 2011, for a review), that consisted in listening to survey or route descriptions and then performing two tasks: a sentence verification task, which tested listening comprehension of spatial relations; and a memory for locations task, in which the children were asked to locate landmarks in an environment based on the description they had heard. In comparing the performance of children with RD and NLD after listening to survey or route descriptions, Mammarella et al. (2009) found that children with NLD had severe difficulty in recalling spatial information based on the presentation of either survey or route descriptions (their difficulty being greater when managing information presented from a survey perspective), whereas children with RD performed adequately by comparison with a TD control group. Overall, these results shed light on the difficulties that NLD children encounter and point to some useful suggestions for the treatment of populations with atypical development (e.g., Broadbent et al., 2014). A limitation of the research conducted by Mammarella et al. (2009) lies, however, in that it was not clear whether NLD children’s difficulties were due to a poor comprehension of the descriptions or to their poor memory. In fact, the tasks (verification and location tests) were performed in a memory condition, i.e., without being able to consult the description while performing the task – as is typically done in mental model studies (Taylor and Tversky, 1992; Brunye and Taylor, 2008).

The main aim of the present research was thus to examine the difficulties children with NLD or RD have in processing spatial descriptions, by comparison with TD controls. In particular, spatial descriptions were presented from survey and route perspectives. After listening to the description, the three groups performed the following tasks: (1) a verification test, answering questions on the spatial relations between landmarks (from the perspective learnt) in two conditions: (i) in a text-present condition (TP), they could refer to the text while answering spatial questions; (ii) in a text-absent condition (TA), the text was withdrawn, thus imposing an additional memory load in the encoding and retrieval phases (e.g., Borella et al., 2011); (2) a location task, in which the children had to locate landmarks graphically in an environment (without consulting the description).

The verification test was administered before the location task to reinforce the encoding of information, which can consequently influence the recall of spatial information. The manipulation introduced by performing the verification test in the TA or TP condition helps to clarify whether the difficulty experienced by children with learning disabilities (and NLD in particular, as shown by Mammarella et al., 2009) in handling spatial information is due to the need to understand it (as tested in the TP condition) and/or memorize it (as tested in the TA condition). The location task also enables us to see whether the verification test condition (TP vs. TA) influences final spatial recall.

It thus seems crucial to separate the two conditions in order to detect different types of difficulty in children with NLD and RD. Since children with NLD have difficulty in processing spatial information (Mammarella et al., 2006) and in recalling spatial descriptions (Mammarella et al., 2009), we expected them to perform poorly in both the TP and the TA conditions. This effect could be seen when testing the description on line (in the verification test) or off line (in the location task). We expected to see different results for children with RD: while in the TA condition they might perform worse than typically-developing (TD) children because of their impaired verbal WM and long-term memory (Kramer et al., 2000), the TP condition might facilitate the RD children (compared to the NLD children, at least) during the verification test. Finally, we expected to find a route perspective offering an advantage over a survey description in the accuracy of all three groups (as suggested by studies on typically- developmental; Ondracek and Allen, 2000; Uttal et al., 2006), NLD having particular difficulty by comparison with RD and TD children (as suggested by Mammarella et al., 2009).

## MATERIALS AND METHODS

### PARTICIPANTS

Thirty-nine children aged 9–12 years were divided into three groups. Twelve (eight boys and four girls, mean age 124.50 months, SD = 26.63) had received a clinical diagnosis of NLD and 12 (10 boys and two girls, mean age 126.75 months, SD = 24.17) had been diagnosed with reading disability (RD), i.e., dyslexia, at the Learning Disabilities Center of the University of Padua (Italy). Fifteen control children (mean age 117.07 months, SD = 2.57) were TD 4th- and 5th-graders attending local schools, matched with the two clinical groups for age, schooling and socio-economic status, but reportedly with no academic difficulties.

Although the NLD and RD children had been diagnosed clinically by a center specializing in learning disabilities, we made sure that the groups met the following criteria. In particular, as recommended in a recent review (see Mammarella and Cornoldi, 2014), the inclusion criteria for the NLD group were as follows: (1) a diagnosis of NLD; (2) a difference of at least 15 points between verbal and perceptual/visuospatial intelligence, i.e., a higher score for the verbal comprehension index (VCI) than for the perceptual organization/perceptual reasoning index (POI/PRI) on the WISC-III or IV scales (Wechsler, 1991, 2003); (3) visuo-constructive difficulties (i.e., <30th percentile in a visual motor integration test); (4) good reading decoding skills (i.e., around average performance for speed and/or accuracy on reading aloud when compared with the normative sample).

The inclusion criteria for the RD group were based on the National Recommendations (AA.VV., 2009) requiring that the child have an average intelligence and a performance below two negative SDs or the fifth percentile in either speed or accuracy when reading aloud.

We ensured that the children included in the two groups were positive for none of the following: (1) treatment with psychoactive drugs; (2) fulfillment of the diagnostic criteria for clinically significant autism spectrum disorder, developmental coordination disorder, or traumatic brain injury; (3) a history of seizures in the previous 2 years; (4) total IQ < 80; (5) poor (or disadvantaged) socio-economic conditions; and (6) medical illness requiring immediate treatment.

All the children spoke Italian as their first language, and none were primarily visually or hearing impaired, or identified as having any neurologically degenerative condition. A signed consent form was obtained from parents and an assent form from each child. In the case of the TD group, consent was given only for the experimental test, not for the other assessments.

### SCREENING TESTS

The screening procedures included the WISC battery (WISC-III, Wechsler, 1991; WISC-IV, Wechsler, 2003), the MT battery (Cornoldi and Colpo, 1981) for measuring children’s reading decoding and reading comprehension skills, and the visual motor integration test (VMI; Beery and Buktenica, 2004) on their visuo-constructive abilities. These tests were administered to all the children with NLD and RD to ensure that the two groups met the above-mentioned criteria. In particular, the MT battery was used to obtain a measure of the children’s reading speed by computing the mean number of syllables read by the child while reading texts aloud (this measure is considered the best indication of a RD for transparent languages). The other two measures obtained with the MT battery concern the number of errors the children made (accuracy) while reading aloud, and the number of correct answers they gave in a reading comprehension task with no time constraints that involved answering multiple-choice questions on the meaning of a passage read silently by the children and remaining available to them at the time of the test.

These screening measures were used for group matching purposes. In particular, children with NLD or RD were matched on the verbal comprehension index (VCI) of the WISC scale, and on the reading comprehension task.

### MATERIALS AND ASSESSMENT MEASURES

#### Spatial descriptions

Participants were presented with eight descriptions of outdoor environments concerning four types of environment (a zoo, an amusement park, a nature park, and a playground), four presented from a route perspective, and four from a survey perspective (adapted from Taylor and Tversky, 1992; and adapted in Italian by Mammarella et al., 2009; Meneghetti et al., 2011). Each spatial description was eight sentences long (with around 200 words altogether) and mentioned five landmarks positioned one in each of four corners and one in the center of an environment in the shape of a square. The equally good recall of the four environments and their landmarks was assessed by means of a pilot study.

In the survey descriptions, the child had to imagine flying over the environment in a helicopter; the description introduced the general layout of the environment, then defined the relations between landmarks within the environment, using terms such as “north,” “south-east,” etc., and presenting the information in a south-to-north direction. In the route descriptions, the child had to imagine walking along a path and the positions of the various landmarks were defined from the child’s point of view using terms such as “left,” “right,” etc. The path described started in the left-hand (zoo and nature park) or right-hand (amusement park and playground) corner of the first side of the environment (the entrance gate). Then the landmarks in the other three corners were mentioned, specifying which side and corner they were on. A preliminary check was run to ensure that the children had a clear idea of the compass points and the right/left sides. For each landmark, non-spatial information was provided to characterize the landmark (e.g., “… the café where you can get very good ice-cream,” “… the cage with the monkeys, who are having fun playing together”). Survey and route descriptions were recalled equally well (as tested previously in a pilot study).

Immediately after presenting each text, a *sentence verification task* was administered, consisting of 6 true/false sentences concerning spatial relations from the same perspective as the description presented. The verification task was used in two conditions: in one (TP) the text remained available to participants when they answered the questions; and in the other (TA) it was withdrawn. One point was awarded for each correct answer, and the maximum score for each description was six.

After completing the verification task, participants were administered a *memory for locations task*: for each environment previously described, the children were given a sheet of A4 paper (29.5 × 21 cm) marked with the square perimeter of an environment and a list of the five landmarks it contained, and they were asked to place the landmarks in their appropriate positions. This task was completed without being able to refer to the description (irrespective of whether the TA or TP condition was used in the verification test). In the survey version of the location task, a compass was drawn on the page to indicate the cardinal points (north, south, east, and west), while in the route version the sides of the square perimeter were labeled (“side 1,” “side 2,” “side 3,” “side 4”). To score the answers given in the location task, two points were awarded for each precisely located landmark (as in Mammarella et al., 2009), i.e., for landmarks correctly placed exactly in the corner or at the center of the environment, and one point for each partially located landmark. In the survey description, a landmark was considered as having been partially located when only a part of the spatial information provided had been taken into account (for instance, in the case of a landmark in the north–east, if the child placed it on the northern or eastern side of the square rather than exactly in the north–eastern corner). Similarly, in the route description, a landmark was judged to have been partially located when the child placed it along either side of the square adjacent to the right corner. A score of 0 was assigned for landmarks located in the wrong positions. For both spatial descriptions, the maximum score was 10.

### PROCEDURE

Children were tested individually. They were presented with four descriptions (one from a route and one from a survey perspective for both the TP and the TA conditions) during a single session lasting approximately 60 min. They were asked to listen twice to each description and memorize the spatial information presented. The text was read to the children slowly (at a rate of ∼2 syllables per second) so that even the children with a RD could follow the reading on the page. In the TP condition, the texts remained available to participants while the experimenter read the text and also when they answered the questions in the verification test, while in the TA condition the text remained available to participants while the experimenter was reading but not when they answered the questions. The order of presentation of the text condition (TP before TA) and the type of task (the sentence verification task before the memory for locations task) was fixed. The task always started with the TP condition because it is more similar to everyday reading situations, as recommended in other studies (e.g., Borella et al., 2011, 2014), and also to limit the use of memory strategies in TP (had the TA condition been presented first). On the other hand, the two perspectives (route and survey) in the various combinations with the types of environment (zoo, amusement park, nature park playground) were counterbalanced. A child might hear a description of the zoo from a route perspective followed by a description of the amusement park from a survey perspective, while another child might hear first about the amusement park from a route perspective and then about the zoo from a survey perspective, and so on.

For the memory for locations task, the children were given the written list of the five landmarks and they had to locate each landmark in the perimeter box drawn on a sheet of paper. There were no time limits for completing the task. As the location task involved locating landmarks, and could influence performance in other tasks, it was always presented after the verification test (see Gyselinck and Meneghetti, 2011).

## RESULTS

### SCREENING TESTS

One-way ANOVAs were run to compare the characteristics of the two groups with disabilities. For the experimental tasks, mixed ANOVAs 3 (Group: NLD vs. RD vs. TD) × 2 (Perspective: route vs. survey) × 2 (Condition: TP vs. TA) were run, and *post hoc* analyses were corrected with Bonferroni’s adjustment for multiple comparisons.

**Table 1** summarizes the IQs and reading and visuo-constructive performance of the children in the NLD and RD groups. The NLD group performed worse than the RD group in terms of FSIQ (full-scale intellectual quotient), *F*(1,22) = 5.45 *p* = 0.029 η^2^ = 0.19, and POI/PRI, *F*(1,22) = 35.80 *p* = 0.0001 η^2^ = 0.62, while the two groups did not differ in VCI, *F*(1,22) = 1.65 *p* = 0.21 η^2^ = 0.07.

**Table 1:** Verbal and non-verbal abilities of children with non-verbal learning disabilities (NLD), and reading disabilities (RD).

	NLD	RD
Characteristics	*M* (SD)	*M* (SD)
**General cognitive skills**
FSIQ^a^	94.25 (10.21)	106.33 (14.74)
VCI	110.33 (13.38)	103.83 (11.36)
POI/PRI^a^	83.08 (12.78)	113.33 (11.97)
**Visuo-constructive skills**
VMI test (percentiles)^a^	16.17 (3.81)	51.17 (8.05)
**Reading abilities**
Speed (z-scores)^b^	-0.36 (0.84)	-2.14 (0.34)
Accuracy (z-scores)^b^	-0.12 (0.66)	-1.44 (0.44)
Comprehension (z-scores)	0.17 (0.47)	-0.11 (0.52)

*FSIQ, full-scale intellectual quotient; VCI = WISC, verbal comprehension index; POI/PRI = WISC, perceptual organization index, perceptual reasoning index; VMI, visual-motor integration test.*

*^a^NLD < RD (*p* ranged from 0.029 to 0.0001); ^b^RD < NLD (*p* = 0.001).*

In the VMI, NLD children performed significantly worse than RD children, *F*(1,22) = 185.15 *p* = 0.0001 η^2^ = 0.89, whereas the RD group performed worse than the NLD group on reading speed, *F*(1,22) = 45.67 *p* = 0.001 η^2^ = 0.68, and accuracy, *F*(1,22) = 33.78 *p* = 0.001 η^2^ = 0.61. The two groups did not differ in reading comprehension *F*(1,22) = 1.80 *p* = 0.19 η^2^ = 0.08.

### VERIFICATION TASK

The 3 × 2 × 2 mixed ANOVA (Group × Perspective × Condition) interaction showed a main significant effect of Group, *F*(1,36) = 6.29 *p* = 0.005 η^2^ = 0.26: the NLD and RD groups both performed worse than the TD children (NLD vs. TD *p* = 0.011; RD vs. TD *p* = 0.018). The Perspective × Group interaction was also significant, *F*(2,36) = 5.87 *p* = 0.006 η^2^ = 0.25 (see **Table 2**), showing that the NLD group was impaired in the case of survey descriptions. In fact, *post hoc* analyses with Bonferroni’s correction showed that the TD (*p* = 0.13) and RD (*p* = 0.36) groups did not differ in the scores they obtained for route and survey descriptions, whereas the NLD group did worse in the verification task when the sentences referred to survey descriptions (*M* = 3.21) as opposed to route descriptions (*M* = 4.21; *p* = 0.003).

**Table 2:** Descriptive statistics for NLD, RD, and TD children’s accuracy in the verification test [in the text-present (TP) and text-absent (TA) condition] and in the location task (performed after completing the verification test in the TP and TA conditions) for survey and route descriptions.

	NLD		RD		TD	
	Route description *M* (SD)	Survey description *M* (SD)	Route description *M* (SD)	Survey description *M* (SD)	Route description *M* (SD)	Survey description *M* (SD)
Verification test – TP condition	4.25 (1.48)	3.42 (1.31)	4.08 (1.51)	3.17 (1.74)	4.87 (0.83)	5.27 (0.88)
Verification test – TA condition	4.17 (1.26)	3.00 (1.65)	3.75 (1.61)	4.93 (1.28)	4.47 (1.06)	4.08 (0.90)
Location task – TP condition	5.92 (3.47)	6.67 (2.64)	7.33 (2.81)	8.33 (1.72)	8.73 (2.02)	9.27 (1.03)
Location task – TA condition	5.58 (3.53)	6.33 (2.61)	5.00 (3.30)	6.83 (2.21)	8.60 (1.81)	8.60 (2.03)

### LOCATION TASK

**FIGURE 1:**
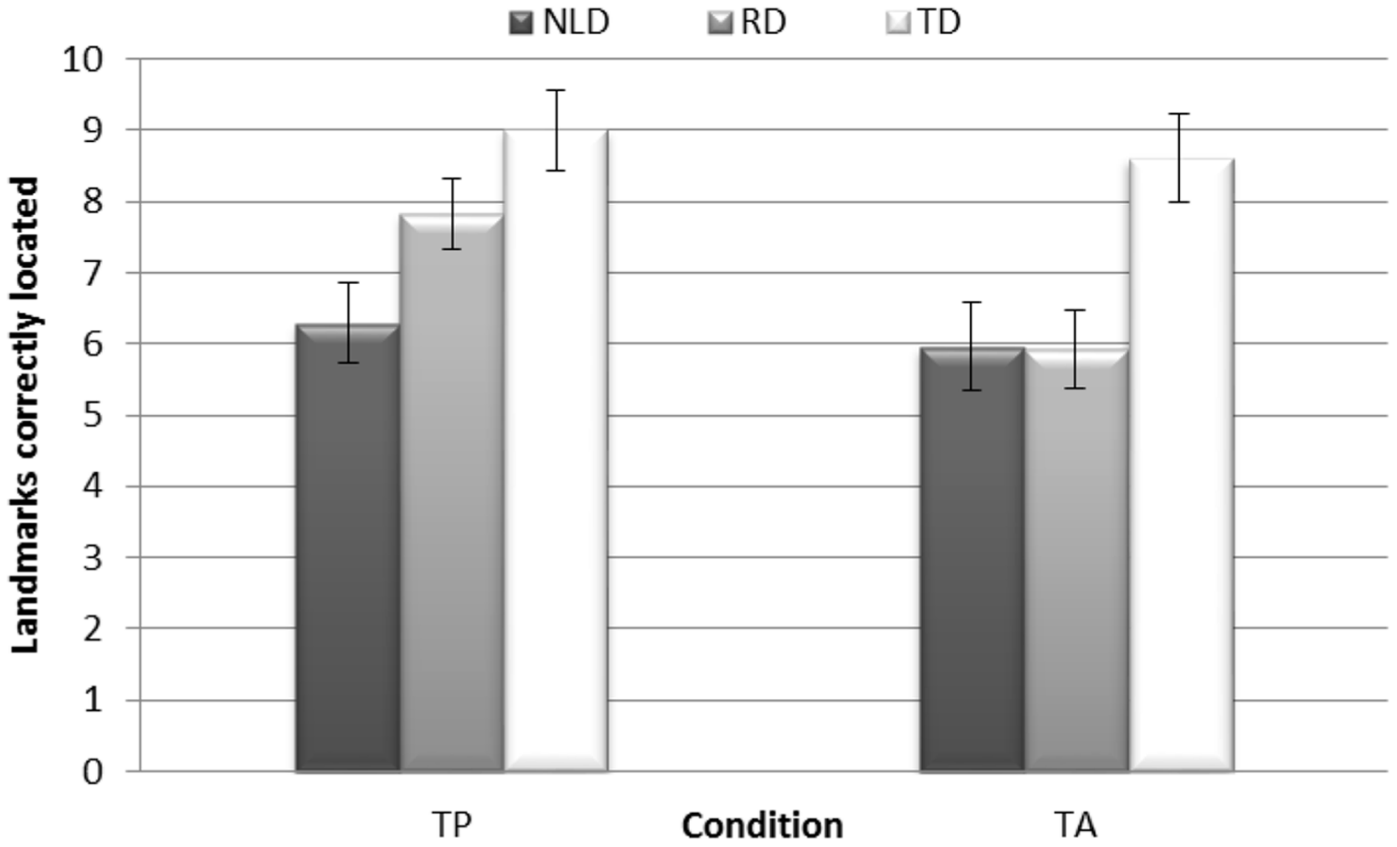
**Location task.** Mean scores for landmarks located correctly (maximum score 10; 2 points for each landmark) by the groups (NLD vs. RD vs. TD) in text-present (TP) and text-absent (TA) conditions. Error bars represent standard errors. TP condition: NLD < TD (*p* = 0.003); TA condition: NLD and RD < TD (*p* = 0.009 and *p* = 0.008, respectively).

The 3 × 2 × 2 mixed ANOVA (Group × Perspective × Condition) showed main significant effects of Group, *F*(2,36) = 4.7.39 *p =* 0.002 η^2^ *= 0*.29, and Perspective, *F*(1,36) = 4.73 *p* = 0.036 η^2^= 0.12, due to performance being better for route descriptions (*M* = 7.67) than for survey descriptions (*M* = 6.86), and also of Condition, *F*(1,36) = 12.15 *p* = 0.0001 η^2^= 0.25, performance in the TP condition being better (*M* = 7.71) than in the TA condition (*M* = 6.82). Concerning the Group effect, both the NLD and the RD groups performed significantly worse than the TD children (NLD vs. TD *p* = 0.002; RD vs TD *p* = 0.036), but the RD group’s difficulty related to the TA condition, as shown in **Figure 1** and **Table 2**. In fact, the Condition x Group interaction was significant, *F*(2,36) = 4.02 *p* = 0.027 η^2^= 0.18. *Post hoc* analyses with Bonferroni’s correction showed that children with NLD performed significantly worse than the TD controls in the TP condition (*p* = 0.003), while the RD children did not differ from the NLD or the TD group; in the TA condition, on the other hand, both the NLD and the RD children performed significantly worse than the TD controls (NLD vs. TD *p* = 0.009; RD vs. TD *p* = 0.008); and only the RD children performed significantly better in the TP than in the TA condition (*p* = 0.0001; see **Figure 1**).

## DISCUSSION

This study aimed to investigate the type of difficulty children with learning disability (RD and NLD) encounter, by comparison with TD children, in processing and remembering spatial descriptions of environments. In particular, we examined whether RD and NLD children differ in their processing and/or recall of spatial information conveyed verbally by spatial descriptions from survey and route perspectives.

To approach this question the study design was as follows: three groups of children – NLD, RD, and TD – listened to survey and route descriptions and then answered true/false spatial sentences. In one condition (TP) they could refer to the text throughout the task, while in the other (TA) the text was withdrawn before they read the sentences. After completing the sentence verification task, the children performed a memory for locations task (without consulting the description) to assess their configured-like knowledge. This type of manipulation clarifies the role of comprehension and memory in spatial description recall.

Judging from our results, the TP condition did not facilitate performance in the verification test, whereas it did help in the location task. In other words, the TP condition improved recall accuracy when participants performed the location task, having had the description available while performing the verification test. These results indicate that the verification test reinforces the encoding of information, and consequently influences the ability to recall spatial information.

This advantage of the TP condition depends on the type of learning disability involved. Children with RD performed better in the TP than in the TA condition in the location task. These results support the impression that children with RD may have long-term memory problems with verbally presented material (Kramer et al., 2000). Conversely, children with NLD had memory difficulties in both the TP and the TA condition, and this goes to show their difficulty in both understanding spatial content (as shown by a poor performance in the verification test) and recalling it (as shown by their performance in the location task). This finding further supports the hypothesis that, despite their good verbal abilities, children with NLD may have language comprehension difficulties (Rourke and Tsatsanis, 1996), especially when it comes to spatial content and they need to generate a spatial model in order to make appropriate inferences (Worling et al., 1999; Mammarella et al., 2009). Although the TP condition revealed differences between the RD and NLD groups, the TA condition imposed an additional verbal memory demand that negatively influenced the performance of both learning disabled groups to much the same extent. These results mean that learning disabilities may be associated not only with WM problems, as shown in an impressive series of studies (see O’Shaughnessy and Swanson, 1998, for a meta-analysis), but also with long-term memory difficulties. Moreover, it was only in the verification test that we found an effect of perspective on the whole sample and by group. In fact, we found that all participants had more difficulty with survey descriptions than with route descriptions, in agreement with the Siegel and White (1975) model, confirming that a survey perspective represents a developmentally more mature stage of environment knowledge acquisition. Previous studies on TD children showed that, after learning spatial descriptions, children formed mental representations with sequential properties from their own point of view, and only later became able to form mental representations with configured-like properties (see Ondracek and Allen, 2000; Uttal et al., 2006). This difficulty with the survey perspective was also more obvious in children with NLD, as shown by the Perspective by Group interaction in the present study, confirming earlier findings of Mammarella et al. (2009).

The fact that children with NLD were weaker with survey descriptions suggests that these descriptions rely on spatial processes more than route descriptions do. In fact, people with good verbal skills but poor spatial skills may find it easy to process route descriptions involving the same sequential structure as the surface structure of language. Taking the mental model approach (Mani and Johnson-Laird, 1982), we could say that NLD children are only able to construct a text-based representation, while RD children can construct a mental model that represents not only the local relationships between landmarks explicitly expressed in the description, but also implicit relationships. Overall, our findings enable us to distinguish between the processes involved in developing and maintaining spatial models in children with different types of learning disability.

As previously mentioned, our sentence verification and memory for locations tasks presumably involved different processes, since the NLD and RD children’s performance revealed different patterns. Children with NLD seemed to have a general comprehension difficulty (and a consequent recall difficulty) when faced with spatial descriptions; while the children with RD seemed better able to understand and process verbally presented spatial relationships, but they needed extra processing for adequate memory encoding, as shown by their better performance in the TP than in the TA condition. These results add to those obtained by Mammarella et al. (2009) in RD children, who did not differ from controls when performing a sentence verification test (in which only the text-absent condition was assessed).

Importantly, our findings need to be confirmed and expanded in future studies. For instance, a limitation of the present study lies in that the role of WM was not tested directly. Previous research has demonstrated the role of VSWM in encoding spatial descriptions (Noordzij et al., 2004; Meneghetti et al., 2013), and the role of verbal WM in processing texts (see Carretti et al., 2009 for a meta-analysis). Previous studies have also documented VSWM deficits in children with NLD (Cornoldi et al., 1995; Mammarella and Cornoldi, 2005a,b) and verbal WM deficits in children with dyslexia (Palmer, 2000; Helland and Asbjørnsen, 2004). The inclusion of WM measures should therefore elucidate to what extent weaknesses in processing spatial texts are due to WM deficits.

Other limitations relate to the procedure used in the present study. We did not use non-spatial sentences as a control measure in the verification task. Although the RD and NLD children were matched for reading comprehension using a standardized screening test, further studies should explore possible differences when spatial and non-spatial information recall is tested after learning spatial descriptions.

No time constraints were used in our study for the verification or the location tasks; the children could take their time to process the text adequately. Further research should examine the general applicability of the effects reported here by considering the role of variables not taken into account in the present case, such as the time taken to complete the tasks, their order of presentation, and the implications for other types of text.

It is worth noting that the differences between children with NLD and RD may have clinical implications. Specifically, our findings provide information that can point to appropriate treatment decisions for children with NLD, and to the risk of spatial descriptions being used with such subjects without carefully considering their features. On the other hand, the children with RD had difficulties relating to memory encoding (since they had much less difficulty when they were given the means to reinforce the latter). Memory encoding problems presumably correlate with linguistic and verbal WM weaknesses – as demonstrated by previous research showing an impaired storage capacity of the phonological loop in children with RD (Gould and Glencross, 1990; Ackerman and Dykman, 1993; Palmer, 2000; Helland and Asbjørnsen, 2004). Such impairments could therefore be contained by offering children with RD the opportunity for a more in-depth encoding, e.g., by letting them refer to a written text, for instance.

In conclusion, the results of the present study extend those previously obtained by Mammarella et al. (2009), and show that children with NLD have specific difficulties in answering questions concerning verbally presented spatial descriptions (especially if they are presented from a survey perspective), and in constructing a spatial mental model with configured-like features. Their difficulties differed to some degree from those seen in children with RD, who performed less well than TD children in the verification task (like the NLD group), but were better able to locate landmarks when they could refer to a text during the encoding phase. These findings should be borne in mind when it comes to treating these children, and offer new insight on the pattern of difficulties experienced by children with NLD and RD.

## Conflict of Interest Statement

The authors declare that the research was conducted in the absence of any commercial or financial relationships that could be construed as a potential conflict of interest.
